# Exclusive breastfeeding in Sri Lanka: problems of interpretation of reported rates

**DOI:** 10.1186/1746-4358-4-14

**Published:** 2009-11-26

**Authors:** Suneth B Agampodi, Thilini C Agampodi, Avanthi de Silva

**Affiliations:** 1 Department of Community Medicine, Faculty of Medicine and Allied Science, Rajarata University of Sri Lanka, Saliyapura, Sri Lanka; 2 Public Health Volunteer Worker, Anuradhapura, Sri Lanka

## Abstract

Accurate interpretation of reported breastfeeding rates is essential in understanding the true picture of a country's breastfeeding status. In Sri Lanka, where the reported exclusive breastfeeding (EBF) rate among infants aged from 0 to 5 months is 75%, accurate understanding of this rate is of the utmost importance. The danger of misinterpreting the data and assuming that Sri Lanka has achieved a high EBF rate is that health workers begin to believe that no further effort should be made in this area. This is very dangerous as the potential to further improve rates of EBF will not be addressed. We discuss the interpretation of survey data and various definitions used in the relevant literature. We strongly recommend that interpretation of EBF rates should be done only after careful evaluation of the definitions and survey methods used.

## Introduction

Sri Lanka declared the achievement of a 50% improvement in rates of exclusive breastfeeding (EBF) in 2007. According to the Demographic and Health Survey (DHS) of 2007, the rate of EBF among infants aged 0-5 months in Sri Lanka was 75% [1]. This is the highest reported rate for the South Asian region [2] and is well beyond even the ten-year target for some countries in the region. It has been highlighted by Sri Lankan Health Authorities as a major achievement in the child health program of Sri Lanka and was commended by UNICEF.

However, the figure of 75% is being misinterpreted among paediatricians, community physicians and other health workers. The DHS reports that, "among infants 0-5 months of age, the percentage who were exclusively breastfed was 75.5%". Informal discussions with healthcare professionals in Sri Lanka indicate that the most common interpretation of this figure is "75.5% of babies in Sri Lanka are exclusively breastfed (from birth) until the completion of six months". This misinterpretation of the data is likely to be widespread in countries that use similar survey methods and reporting processes.

The purpose of this paper is to discuss the problems of definitions, measurements and interpretation of EBF rates reported in the DHS as it relates to Sri Lanka.

## Discussion

### Definitions for estimating EBF rates

Definitions used in breastfeeding were not agreed upon by most of the researchers for many years. Labbok discussed the need for consistency in breastfeeding definitions in 1990 [3] and called for action in 1997 [4]. In 1988, the first international meeting related to breastfeeding definitions was held, sponsored by the Interagency Group for Action on Breastfeeding (IGAB). This was followed by a set of definitions published by WHO/UNICEF, which was used extensively by researchers. The WHO published an update of these definitions in 2008 [5].

For cross-sectional studies, WHO recommends estimating EBF rates based on the 24-hour recall method. In this method, information is collected on feeding practices for the day (24 hours) preceding the survey. EBF is defined by WHO as "The infant has received only breast milk from the mother or a wet nurse, or expressed breast milk, and no other liquids or solids with the exception of drops or syrups consisting of vitamins, mineral supplements, or medicines" [5].

Another widely used method of estimating EBF in surveys is the use of 'recall since birth', in which an infant is categorised as exclusively breastfed only if they have not received any food or drink other than breastmilk since birth [4]. This method requires a longer recall period, but strictly emphasises the WHO infant feeding definitions since birth.

### Estimating EBF rates in populations

Defining the specific age or age categories is crucial for the measurement of EBF in populations. WHO recommends use of the indicator 'Exclusive breastfeeding under six months', defined as the 'proportion of infants 0-5 months of age who are fed exclusively with breast milk' based on the 24-hour recall method [6].

The age range for this method is from birth to just under six months of age (0-5.99 months). It is important to note that both numerator and denominator include infants in all ages within the given range and does not represent the proportion of infants who are exclusively breastfed until just under 6 months of age. However, WHO recommends further categorization of this age range as 0-1, 2-3 and 4-5 months age groups, so that the rates are specific to the given age range.

### Interpretation of survey results

Since there are various definitions of EBF and a number of ways in which the surveys can be conducted, interpretation of survey results should be based on the methodology used and the age group of infants included in the study group. The Sri Lankan DHS 2007 reported, "Among infants 0-5 months of age, the percentage of exclusively breastfed infants was 75.5%". This is the average rate of breastfeeding among infants aged 0-5 months. The same survey has reported the breakdown of this rate as age specific rates. According to these rates, among infants aged 4-5 months, the percentage of exclusively breastfed infants was 53.4%. However, in the 0-1 month age group the percentage was 92.2 and the 2-3 months age group it was 83.7% (Table 1). The table clearly shows how EBF declines steadily from birth. Among infants aged 4-6 six months, 46.6% were not exclusively breastfed during the 24-hour period prior to the survey.

**Table 1:** Breastfeeding status in Sri Lanka by age (DHS 2007) [1]

Age in months	Number of children studied	Percentage exclusively breastfed
0-1	172	92.2
2-3	242	83.7
4-5	221	53.4
6-8	325	7.2

### Limitations of the 24-hour recall method

The Sri Lankan DHS uses the WHO recommended 24 hour recall method to estimate EBF rates. The 24-hour recall method can always overestimate the actual EBF rate in a population. In 24-hour recall method, investigators categorize infants who were on infrequent liquids or foods, but not given those foods/liquids during the previous day as exclusively breastfed infants.

Several authors have questioned the validity of 24 hour recall method [6,7] and studies have shown that this method substantially overestimates the EBF rate, compared to the "recall since birth" method [8-10]. One of these papers reports that the difference was as high as 59% at four months [11]. In Sri Lanka, the validity of EBF as determined using the 24-hour recall method was evaluated against the measurement of EBF since birth [12]. According to the Sri Lankan study, the proportion of infants breastfed exclusively using the 24 hour recall method was 77.4% whereas according to recall since birth it was only 49.1%. Sensitivity of the WHO definition to detect non-EBF infants was 42.9% (95% CI: 26.5, 60.9%) while the negative predictive value was 60.1% (95% CI: 45.7, 74.4%). This suggests that the reported rate of EBF of 53.4% for 4-5 months infants using the 24-hour recall method might be an overestimation of the actual rate and the actual percentage of mothers practising EBF for the recommended six months period (EBF since birth to 6 months) in Sri Lanka is well below 50%.

A proper understanding and interpretation of survey results on breastfeeding is very important for healthcare providers to understand the true status of EBF so that they can deliver services accordingly. Accurate interpretation also affects the appropriate design of policies and programs, such as the Sri Lanka Infant Feeding Program and the Child Health Program. Although Sri Lanka has recently seen great improvements in EBF, inaccuracies in interpretation have led to the belief that deficiencies in EBF in the population have been sufficiently addressed and that best practices have been achieved. In fact at least 46.6% of infants are not being exclusively breastfed at 4-5 months of age. Rates of EBF from birth to the completion of 6 months (0-5.99 months) is likely to be as low as 20% [13,14]. Further, the evaluation of definitions showed very low sensitivity of the 24-hour recall method to detect non-EBF infants.

The danger of misinterpreting the data and assuming that Sri Lanka has achieved a high EBF rate is that health workers begin to believe that no further effort should be made in this area. This is very dangerous as the potential to further improve rates of EBF will not be addressed, thereby overlooking an important opportunity to advance child health. It should also be noted that improving EBF in Sri Lanka is not a costly and difficult process, as aptly demonstrated by a study conducted in the Medical Officer of Health Area, Beruwala, Sri Lanka [15].

## Conclusion

We strongly recommend that interpretation of EBF rates should be done only after careful evaluation of the definitions and survey methods used. As the duration of EBF has been widely discussed over the past few years, a consensus of methods to properly evaluate this indicator should be reached. Programs to strengthen EBF in Sri Lanka remain a priority as the actual rate of EBF to six months in the country is well below the often quoted, but misinterpreted, rate of 75%.

## Competing interests

The authors declare that they have no competing interests.

## Authors' contributions

All authors participated in literature review, conceptualization and drafting of the manuscript and read and approved the final manuscript.

## Authors' information

SBA and TCA are lecturers in Community Medicine in Rajarata University of Sri Lanka with Masters in Community Medicine. Both worked as Community Physicians and have experience in conducting breastfeeding research, promotion and training. SBA is currently a doctoral student and attached to the Post Graduate Institute of Medicine, Colombo, Sri Lanka. AdS is a BSc graduate in Biomedical Science and a public health volunteer worker in the field practice area of the Faculty of Medicine and Allied Sciences, Rajarata University of Sri Lanka.
